# The feasibility and impact of implementing a computer-guided consultation to target health inequality in Asthma

**DOI:** 10.1038/s41533-023-00329-8

**Published:** 2023-02-07

**Authors:** B. Chakrabarti, B. Kane, C. Barrow, J. Stonebanks, L. Reed, M. G. Pearson, L. Davies, M. Osborne, P. England, D. Litchfield, E. McKnight, R. M. Angus

**Affiliations:** 1 Liverpool University Hospitals NHS Foundation Trust, Liverpool, UK; 2 Health Innovation Manchester, Manchester, UK; 3 LungHealth Ltd, Swaffham, UK

**Keywords:** Asthma, Health policy

## Abstract

Greater Manchester has a greater prevalence and worse asthma outcomes than the national average. This study aims to evaluate a digital approach to primary care asthma management and in particular the initial impact of implementing Clinical Decision Support System software in the form of a computer-guided consultation (CGC) in the setting of primary care asthma reviews in deprived areas of Greater Manchester. The CGC (LungHealth Ltd) is an intelligent decision support system ensuring accurate guideline-based staging of asthma and assessment of asthma control with the software subsequently prompting guideline-standard management. Patients on asthma registers in Greater Manchester Primary Care Networks were identified and underwent remote review by nursing staff using the CGC linked directly to the GP clinical system. Three-hundred thirty-eight patients (mean age 59 (SD 17) years; 60% Female) were reviewed. The CGC reported the patient’s asthma control to be “Good” in 22%, “Partial” in 6% and “Poor” in 72%. ACT scores were significantly higher in those patients exhibiting “Good” and “Partial” control when compared to those with “Poor” control. The number of steroid courses and hospital admissions in the previous 12 months was significantly lower in those patients exhibiting “Good” and “Partial” control when compared to those with “Poor” control. Nineteen percent were found not to have a personalised asthma management plan during CGC review, which was alerted by the CGC and subsequently, all but 3 patients had this created on review completion (McNemar’s test; *p* < 0.001). 5% were found not to have been prescribed regular inhaled steroid therapy resulting in the operator being alerted by the CGC in all cases. Overall, 44% underwent alteration in asthma therapy following the CGC review with 82% of these representing treatment escalation. An end-to-end digital service solution is feasible for Asthma within primary care and the utilisation of a CGC when conducting primary care asthma reviews increases implementation of guideline-level management thus addressing healthcare inequality while enabling identification of “high risk” asthma patients and guiding appropriate therapy escalation and de-escalation.

## Introduction

Addressing healthcare inequality is a major priority for NHS England as described in the Core20PLUS5 initiative where a key priority is chronic respiratory disease^1^. Asthma is a major cause of morbidity and avoidable healthcare utilisation in the United Kingdom with greater prevalence and worse outcomes in the more deprived areas of the country. Four of the most deprived local authorities in terms of healthcare outcomes are in Greater Manchester, which has a below average life expectancy and a greater asthma prevalence with poorer outcomes than the England average^2^. This is indicated by a higher emergency hospital admission rate and some of the highest rates of over-reliance on short acting beta agonist (SABA) medication when compared to other Sustainability and Transformation Partnerships (STPs)^3–5^.

There have been a number of national asthma audits since 1963^5–11^ but despite the widespread availability of evidence-based guidelines since the 1990s the findings and recommendations from these audits have remained unchanged with little evidence of improvement in care or outcomes. Common themes emerging from these audits include a failure to recognise asthma severity and to follow recognised clinical guidelines. This includes the under-prescribing of inhaled corticosteroids (ICS), inadequate utilisation of personal management plans and a lack of timely specialist referral where clinically indicated. Each subsequent audit has highlighted similar recommendations including improved recognition of the severity/risk of disease in individual patients, a structured clinical assessment, better use of physiological measurements, earlier and more consistent use of inhaled corticosteroids, patient education including written personal action plans, more robust follow-up along with involvement of specialist care when required and better adherence to asthma guidelines when prescribing. Thus, simple dissemination of such paper-based guidelines has not proved to be an effective strategy in improving patient outcomes. Indeed, the National Review of Asthma Deaths (NRAD) report from 2014 mirrored the findings of the first UK asthma deaths report more than 50 years earlier highlighting the challenge of how to improve asthma care and outcomes, particularly in areas of high deprivation^6–10^.

Health Innovation Manchester, an academic health science and innovation system, was formed with the aim of bringing together health and care, industry and academia to accelerate innovation and improve the health and wellbeing of Greater Manchester’s 2.8 million citizens by addressing challenges and tackling inequalities. Asthma is a priority as the Greater Manchester region carries a significant burden in the form of health inequality, which is reflected in a high number of emergency hospital admissions and significant morbidity due to asthma^10–12^. The Standardising Asthma Reviews and Reducing SABA overuse in Greater Manchester (STARRS-GM) project aims to enhance the outcomes for people living with asthma in the region through proactive identification and reviews of high-risk patients to improve their asthma management. An integral part of the project is the use of technology including a bespoke audit tool to identify patients most likely to benefit from review and the introduction of clinical decision support system software in the form of a clinical guided consultation system (CGC). We have previously reported that the use of a CGC results in greater implementation of guideline-level care in both chronic obstructive pulmonary disease (COPD) and obstructive sleep apnoea (OSA)^13,14^. In this preliminary evaluation, we report the initial impact of implementing these technologies within primary care as part of the STARRS-GM project pathway.

## Methods

### STARRS-GM Clinical pathway

The Standardising Asthma Reviews and Reducing SABA overuse in Greater Manchester (STARRS-GM) project aims to improve patient outcomes in asthma through greater implementation of guideline-level care.

The project aims to determine the reduction in SABA usage and unscheduled healthcare utilisation resulting from the implementation of the pathway. The project aims to identify and subsequently optimise asthma management in “high risk” patients as defined by patients who have received 6 or more SABA inhalers in the previous 12 months and who also had at least one NRAD risk criteria and prompt appropriate specialist asthma multi-disciplinary team (MDT) input^6^. A second group of patients with good asthma control on high-dose inhaled steroid were also reviewed. This group may be on higher levels of treatment than they necessarily require and a targeted review of this group may result in de-escalation of therapy in some patients reducing potential drug side effects for the individuals while releasing resource that could be used elsewhere in asthma care.

**Fig. 1:**
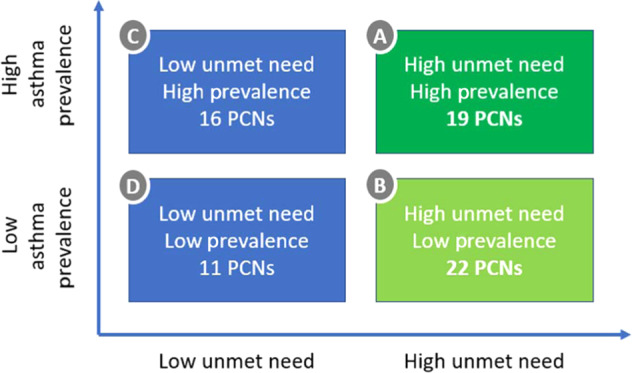
Matching Asthma Need With Prevalence. Grouping of PCNs by unmet need and asthma prevalence in the STARRS-GM project.

Primary care networks (PCNs; referring to a group of primary care practices within a given locality working towards common healthcare outcomes) in the Greater Manchester area were approached to take part in the STARRS-GM project and prioritised based on asthma prevalence and level of unmet need. “High asthma prevalence” was defined as >2600 asthma patients on the Quality and Outcomes Framework (QOF) register, while “high unmet need” was defined as 60+ percentile SABA use as a proportion of total SABA plus inhaled corticosteroid use when compared to all other PCNs in England. In order to meet the Core20PLUS5 agenda PCNs in groups A and B were prioritised (Fig. 1)

To meet the objectives of the STARRS-GM project by reviewing patients either at risk of poor outcomes because of their asthma or those where step down of therapy may be possible, two groups of patients were identified using a bespoke MIQUEST (Morbidity Query Information Export SynTax)^©^/SNOMED (Systematised Nomenclature of Medicine Clinical Terms)^©^ Software tool. GP systems are constructed to allow bespoke searches and this tool examines the GP asthma register pulling out all key disease features including medication, clinical events and review history. The Software collects information on the prescriptions made by the GP, though not those filled by a pharmacy (Box 1).

Here, patient identification was conducted utilising a SNOMED/MIQUEST risk stratification tool followed by a nurse case notes review confirming the patient selection and allocation to a group.

Cohort 1: Patients deemed at “high risk” of adverse asthma outcomes i.e., those collecting 6 or more SABA inhalers in the previous 12 months together with at least one of the following additional NRAD “at risk” criteria highlighted below were identified:

- Hospital admission as a result of their asthma in the last 12 months
- Attendance at out of hours (OOH) and/or Emergency Department (ED) with an asthma exacerbation
- Two or more short courses of prednisolone for asthma in the previous 12 months
- Under-use of preventer medication (defined as <75% of recommendation)
- No recorded inhaler technique or inhaler technique recorded as poor
- No record of an annual review for their asthma

Cohort 2: Patients on high-dose inhaled corticosteroid therapy with all the following criteria were identified as potentially suitable for de-escalation of anti-inflammatory therapy:

- No exacerbations in the previous 12 months
- Asthma Control Test controlled upon last review with a score >19^15,16^
- No hospital admissions in the previous 12 months
- No ED or OOH attendances for asthma in the previous 12 months

Eight practices from 3 PCN’s participated. All asthma patients from the practices were identified, a profile was run and those in the two cohorts began to be invited for asthma consultations using the practices’ standard means of contacting their patients. Consultations were conducted remotely by secure video calls using the standard Accurx© platform. If patients did not have a “smart-phone” or other video capable device, a telephone review was offered. Patients without telephones were identified for a traditional review and have not been included in this report. Patients were reviewed by respiratory trained primary care specialist nurses (National Services for Health Improvement Ltd) utilising an asthma-specific computer-guided consultation (LungHealth Asthma CGC). All patients gave individual consent to review using this CGC and to the holding of their data, including pooled anonymous data to be used for reports and research. The work was discussed with the Health Research Authority who indicated that they regarded this as a service development and that ethics approval was thus not required.

Box 1 Features of the Asthma computer-guided consultation (CGC) Asthma CGC guides the healthcare professional through a number of sections incorporating the following components

- Staging of the patient’s asthma treatment according to the BTS SIGN guidelines (https://www.brit-thoracic.org.uk/news/2019/btssign-british-guideline-on-the-management-of-asthma-2019/)
- Assessment of asthma control using a multi-dimensional algorithmic process taking account of established questionnaire and physiological criteria (e.g. Asthma Control Test, peak flow readings, previous healthcare utilisation) used in combination with control being divided into “Good”, “Partial” and “Poor”
- Identification of key trigger factors (including occupation) for asthma and presence of cardinal “red flags” in the asthma history e.g. history of mechanical ventilation due to asthma
- Assessment of adherence to medications including the functionality to link to the number of SABA inhalers collected by the patient (using the MIQUEST© toolkit) and inhaler technique check
- Recording and intelligent interpretation of key physiological measurements such as Exhaled Nitric Oxide (FeNO) incorporating this into a therapy de-escalation algorithm
- Alerting the operator to a patient meeting NRAD criteria risk factors for future adverse asthma outcomes and highlighting those patients requiring earlier follow up
- Prompting the operator to escalate or de-escalate asthma therapy where appropriate based on key components of the CGC review and prompting need for specialist referral based on BTS SIGN guidelines
- Highlights guideline-based non-pharmacological therapy e.g. formulation of written personalised asthma management plans and discussion of smoking cessation where appropriate

### The LungHealth Asthma computer-guided consultation (CGC)

The CGC (LungHealth Ltd) enables an intelligent structured electronic asthma review. It can be used to review patients remotely or face to face. Using the medical model and constructed to reflect evidence-based guidelines, natural consultations flows are followed but with standardisation. Algorithms are embedded in the software and these prompt supplementary questions and management considerations, which are individualised to every patient dependent upon their response to questions (see Box 1) and may be customised to local guidance priorities such as medicines management. The CGC leads the healthcare professional and the patient through a structured asthma review, asking questions to enable the determination of triggers, asthma control and severity and so leading to prompts around the best treatments (pharmacological and non-pharmacological) for every individual. Although the CGC “suggests” management options, the final decision about how to manage the patient remains with the healthcare professional.

The CGC produces an electronic report that can be written back into the Electronic Health Record (EHR) for the systems commonly used in the UK. In the UK, this also populates the fields necessary for the quality and outcomes framework. The CGC is hosted on a local UK NHS server and has two-way connectivity with the primary care server. Its use is password protected enabling Caldicott principles and General Data Protection Regulations to be satisfied thus ensuring patient data gathered during consultations is duly and lawfully protected and that these data are only used when it is appropriate to do so, with anonymity being preserved^17^ (https://ico.org.uk/for-organisations/guide-to-data-protection/guide-to-the-general-data-protection-regulation-gdpr/).

### Statistical analysis

Statistical analysis was performed using SPSS 28.0. Data are presented as mean ± SD unless otherwise stated. Statistical significance was defined as a *p*-value < 0.05. We used the independent sample *t*-test to identify significant differences in continuous variables and the Chi-squared test for categorical variables. The McNemar’s test was used to determine significant differences on a dichotomous dependent variable between paired data.

### Reporting summary

Further information on research design is available in the Nature Research Reporting Summary linked to this article.

## Results

The eight practices had 76,270 patients on their lists with 4791 identified as having asthma. At the cut point for analysis 338 patients had received a guided consultation. Of them 291 fell into cohort 1, 29 into cohort 2 and 18 patients with asthma but not in the two groups also received a review; the practices confirm the last group were contacted in error.

A total of 338 patients (mean age 59 (SD 17) years; 60% Female) on the GP asthma register in one of the two cohorts described above were identified using the Miquest toolkit and underwent CGC review. CGC review enables the identification of patients according to BTS/SIGN therapy stages^16^. The CGC characterised the patients’ asthma control using ACT/RCP/GINA to be “Good” in 22% (*n* = 75), “Partial” in 6% (*n* = 19) and “Poor” in 72% (*n* = 244). The level of asthma control for patients in each of these BTS therapy stages (https://www.brit-thoracic.org.uk/news/2019/btssign-british-guideline-on-the-management-of-asthma-2019/) is shown in Table 1.

**Table 1:** Asthma control at each BTS/SIGN therapy stage.

Asthma stage by CGC (BTS/SIGN guidelines)	CGC reported “Good” control (*n* = 75)	CGC reported “Partial” Control (*n* = 19)	CGC reported “Poor control” (*n* = 244)
Non-guideline therapy (*n* = 1)	0	0	1
Intermittent reliever therapy i.e., as needed SABA (*n* = 16)	2	2	12
Regular preventer therapy i.e., low-dose ICS (*n* = 48)	14	8	26
Initial Add-On therapy ICS/LABA (*n* = 33)	6	6	21
Additional Controller therapy (*n* = 93)	31	1	61
Specialist Therapies (as per BTS SIGN guideline) (*n* = 147)	22	2	123

The relationship between the CGC definition of asthma control with key multi-dimensional components comprising the assessment of asthma control as well as SABA use is illustrated in Tables 2 & 3.

**Table 2:** Relationship between CGC definition of asthma control and clinical parameters.

	“Good” control (*n* = 75)	“Partial” control (*n* = 19)	“Poor” control (*n* = 244)
ACT score (mean/SD)	23.23 (1.49) (*p* < 0.001)^a^	21.32 (1.60) (*p* < 0.001)^a^	15.71 (4.11)
Number of oral corticosteroid courses in previous 12 months (mean SD)	0 (0) (*p* < 0.001)^a^	0.53 (1.35) (*p* < 0.01)^a^	1.41 (2.34)
Number of hospital/ED visits in previous 12 months (mean SD)	0 (0) (*p* < 0.01)^a^	0 (0) (*p* < 0.01)^a^	1.92 (1.66)

^a^McNemar’s test when compared with poor control.

**Table 3:** Relationship between CGC definition of asthma control and inhaler use.

	“Good” control	“Poor” control	*p*-value
SABA prescribed	7.85 (3.81) (*n* = 74)	9.36 (3.92) (*n* = 240)	*p* = 0.004
SABA reported as used	4.94 (3.55) (*n* = 54)	8.74(5.42) (*n* = 223)	*p* < 0.001
Preventer inhaler prescribed	7.84 (4.35) (*n* = 75)	8.32 (4.19) (*n* = 241)	*p* = 0.37
Preventer inhaler reported as used	7.51 (4.34) (*n* = 74)	8.00 (4.00) (*n* = 237)	*p* = 0.39

The ACT scores were significantly higher in those patients exhibiting “Good” and “Partial” control when compared to those with “Poor” control (*p* < 0.001). The number of oral corticosteroid courses in the previous 12 months was significantly lower in those patients exhibiting “Good” and “Partial” control when compared to those with “Poor” control (*p* < 0.01) and (*p* < 0.001), respectively.

Hospital admissions in the previous 12 months were significantly lower in those patients exhibiting “Good” and “Partial” control (none in both of these groups) when compared to those with “Poor” control (13 patients were admitted to hospital in this group; *p* < 0.001).

Overall, the mean number of SABA inhalers prescribed for the patients was significantly higher compared to the number reportedly used by the patient over a 12-month period (8.92 (SD 3.88) v 7.84 (SD 5.23); 95% CI 0.36 to 1.80; *p* = 0.003). The number of SABA inhalers used in the previous 12 months was significantly lower in those patients deemed to “Good” control by the CGC compared to those deemed to have “Poor” control in those where this data was collected by the CGC (see Table 3). The same relationship was observed in terms of the number of SABA inhalers collected by the patient with a significantly lower number collected in those with “Good” control. The number of preventer inhalers prescribed in the previous 12 months did not significantly differ in those patients deemed to have “Good” control by the CGC compared to those deemed to have “Poor” control.

Review using the CGC highlighted three patients who had previously been intubated and ventilated due to asthma. Despite asthma control currently being “Good” in one of these patients, the CGC flagged up the previous history and alerted the operator that this patient should be considered for specialist follow up.

Table 4 summarises some key outcomes resulting from the CGC review. 66 (19%) patients were identified as having no written personalised action plan and following CGC review, this was achieved in all but 3 patients (McNemar’s test; *p* < 0.001).

**Table 4:** Management changes prompted by CGC.

	Number identified by CGC	Action following CGC review
Absence of a written personal action plan	66 (19%)	63 given personal action plans
No regular ‘Preventer’	17 (5%)	14 prescribed regular inhaled steroid therapy
Inadequate inhaler technique	31 (9%)	21(6%) in whom a spacer was added.
Poor adherence	63 (18.5%)	Importance of adherence and reasons for poor adherence discussed with all
Current smokers	85 (25%)	All prompted regarding Smoking cessation and invited to be referred to local smoking cessation services
Sub-optimal Asthma control at the “Specialist therapies” and “Additional Controller” stage meriting consideration of specialist assessment	184 (77%) had “poor” control	Prompt to consider referral for specialist assessment.

Eighty-five patients (25%) were identified as being current smokers and the CGC prompted nurses to deliver smoking cessation advice for all these patients though only 4 patients agreed to be referred for further support.

Of the 16 patients identified as being prescribed “salbutamol only” and the one patient on a non-guideline regimen (see Table 1), all but 3 patients were started on inhaled corticosteroid therapy following CGC review (McNemar’s test; *p* < 0.001). Of these 16 patients on salbutamol only, mean 12-month SABA inhaler use was 6.00 (SD 4.02) and ACT score 18.38 (SD 3.54) with asthma control deemed by the CGC to be “Poor” in 12 of these 16 patients.

71% (240/340) of patients undergoing CGC review were staged either at “Specialist therapies” or “Additional Controller” stage (see Table 1). The CGC determined asthma control to be “poor” in 77% (184/240) of this sub-group and in all cases prompted the operator to consider referral for specialist assessment.

Overall, CGC review recommended a change in asthma therapy in 44% (149/338) of patients with 82% (*n* = 122) of these changes representing therapy escalation. “Good” control was reported by the CGC in 75 patients (22%). The CGC prompted consideration of therapy de-escalation where appropriate in 73 of these patients with de-escalation not being appropriate in 2 patients as they were on intermittent reliever therapy. Of those 73 patients with “Good control” where the CGC recommended therapy de-escalation, the operator chose actually to de-escalate therapy in 37% (*n* = 27) after discussion with the patient’s GP practice. When taking this “Good control” group, 22 patients were on “Specialist Therapies” of whom 8 were de-escalated and 31 where on “Additional Controller” therapy of whom 14 were de-escalated.

## Discussion

This initial evaluation of the STARRS-GM approach was undertaken to determine the feasibility of this comprehensive digital approach and particularly the utility of the LungHealth asthma computer-guided consultation (CGC). Health informatics and multiple deprivation index metrics were utilised to select one of the most deprived areas and the primary care networks serving Greater Manchester. This allowed the identification of a PCN with the challenge of excessive SABA use and poor asthma outcomes.

In this PCN the bespoke MIQUEST/SNOMED search tool was used to identify two cohorts of patients for review, the LungHealth asthma guided consultation was then utilised. The results show that the approach is practical. When the 338 patients receiving the guide consultation are considered, the first observation is that the CGC characterised patients grouping them into levels of control (as seen in Table 1) suggesting that use of the MIQUEST/SNOMED tool could be used to correctly prioritise selected patients for review using the guided consultation. At this point we recognise that only a proportion of the population has been evaluated. It is possible that in the whole cohort the tool would prove to be less specific, however, this data gave us enough assurance to continue the project with this search methodology. The consultation was also seen to be adept at identifying issues with care, which may lead to excessive SABA use and poor asthma control and identifying gaps in patient care. In addition to identifying and addressing gaps in their care such as 19% not having written action plans or the poor adherence in 18.5%, use of the CGC also prompts medication changes towards guideline management, though the healthcare professional does make the final decision as described. 44% of those reviewed had medication changes recommended with a step up in 82% and a step down in 18%. Referral for specialist assessment was also suggested in a significant number of patients though it must be noted that the population studies here is a subset of those on asthma register and many patients were selected for review because they were identified as being poorly controlled.

In primary care services, healthcare professionals are faced with the challenge of implementing an increasing number of complex clinical guidelines from different specialties to deliver optimal patient outcomes^18^. However, despite an emphasis on the importance of guideline-standard care, it is apparent that in conditions such as asthma the strategy of guideline dissemination in the hope of this translating into clinical benefit has yielded limited success. For example, while it is evident that the use of written personalised action plans and patient education leads to a significant reduction in healthcare utilisation, the implementation of this key practice point has been historically low, a finding mirrored here where 19% of patients were lacking a personalised action plan^19,20^. However, following CGC review, this had been achieved for nearly every patient in this cohort suggesting that the introduction of such intelligent clinical decision support system software into patient pathways may lead to a greater uptake of evidence-based practice, upskilling healthcare professionals and reducing variation in the delivery of care as has been demonstrated previously in the setting of COPD and OSA^13,14^. The CGC assesses asthma control using a multi-dimensional framework incorporating validated tools such as the ACT, assessment of adherence and physiological indices such as lung function and its algorithms also prompt the operator to consider asthma triggers and suspected occupational factors during review. All this ensures that patients with symptoms of uncontrolled asthma are not missed during a CGC consultation and are highlighted to the operator for further action. The National Review of Asthma Deaths stressed the need for patients to adhere to regular inhaled corticosteroid medication in order to maintain good asthma control and prevent deaths^6^. The use of the CGC highlighted 5% of patients who were found not to have been prescribed regular inhaled corticosteroid therapy despite the majority of this sub-group having poorly controlled asthma at the time of review. Following CGC review, all but one of these patients were commenced on regular inhaled corticosteroid therapy thus reducing the risk of future harm due to uncontrolled asthma. The finding of excess SABA use in a patient also represents a risk factor for future asthma attacks and national guidance states that the identification of this future risk is an important component in the delivery of personalised asthma care^6^ (https://www.brit-thoracic.org.uk/news/2019/btssign-british-guideline-on-the-management-of-asthma-2019/). Meeting this requirement is an area integral to CGC functionality as its algorithms alert the operator to those patients who meet guideline thresholds for excess SABA use and inhaled corticosteroid underuse.

Another important deficiency in asthma care that has come under recent scrutiny concerns the failure of healthcare professionals to recognise severe asthma in a timely and appropriate manner and trigger referral for specialist assessment according to guideline-based practice. This is particularly apparent with the advent of biologic therapies^21–23^. The implementation of the CGC resulted in three quarters of the cohort in the “specialist therapies” stage” or at the “additional controller” stage being identified as sub-optimally controlled. The CGC works to prompt specialist referral in such cases while also taking into account other modifiable factors such as adherence and any acute precipitating factors. At the opposite end of the spectrum, there remains a reluctance to de-escalate treatment in asthma where it is safe and clinically appropriate to do so thus risking adverse clinical and health economic consequences, e.g., side effects of high-dose inhaled corticosteroids^24^. The CGC prompted consideration of de-escalation in most cases where it deemed asthma control to be “good” with the operator actually de-escalating therapy in 37% of these cases. A 6-month prospective Dutch study focusing on severe asthma demonstrated that encouragingly, the use of an internet-based tool incorporating fractional exhaled nitric oxide (FeNO) levels and asthma control questionnaire (ACQ) resulted in a reduction in steroid dose (median cumulative steroid dose was 205 mg lower in the intervention group) without a deterioration in asthma control^25^. Our evaluation did not utilise FeNO measurements when stepping down therapy on this occasion but did reveal a significant difference between the number of reliever inhalers collected and those actually used. While these data are limited by self-reporting actual inhaler usage, it raises the important issue regarding the health economic impacts of medicines wastage and encourages development of strategies to address this issue^26^.

The role of clinical decision support software (CDSS) in the assessment of adult asthma in the UK has been described previously in the literature^27,28^. A Canadian study reported the impact of CDSS software on the uptake of asthma action plans and reported an increase in uptake from 0 to 17.8% and an increase in the proportion undergoing assessment of asthma control with a proportion of patients having therapy escalated compared^27^. One difference between the CDSS evaluated by these authors and that reported here is that in the latter, assessing asthma control is mandatory in order to complete the consultation. A critique of CDSS applicability in asthma published in 2014 commented that the effectiveness of such technology was found to be limited at the time due to the system’s recommendations not always being followed and a paucity of use^28^. However, since then, the increasing imbalance between capacity and demand within healthcare systems alongside the challenges posed by the COVID-19 pandemic has created new opportunities for the development and evolution of such digital solutions particularly when systems are fully integrated within the primary care EHR as in the case of the CGC reported here. Importantly, the remote capability of the CGC coupled with direct two-way connectivity to the primary care server enables elective primary care reviews to continue during pandemic conditions as patients may undergo such reviews from home and indeed healthcare professional can also work remotely if required.

This service evaluation carries some limitations in terms of extrapolation to wider clinical practice. All patients undergoing review with the CGC were on the GP asthma register with a primary care diagnosis of Asthma. It is recognised that there are patients on primary care Asthma registers who may not have a true diagnosis of Asthma and this evaluation does not take such a cohort into account^29^. However, the CGC is currently being further developed to consider important differential diagnoses and the presence of atypical symptoms in patients with a less certain asthma diagnosis. Further studies are required in this area to determine diagnostic validity in this setting.

The importance of appropriate use of and adherence to asthma medications cannot be overemphasised in clinical practice. The implementation of this CGC with the existing linkage to the primary care server and the MIQUEST© tool enables those patients who are deemed at being high risk of adverse asthma outcomes (e.g., excess SABA use and underuse of inhaled corticosteroids) easily to be identified and invited for a structured CGC review. Where poor adherence was addressed by patient education on the benefit of regular medicines, reinforcing self-management, addressing inhaler technique and arranging earlier follow up. However, at present, any benefit of the CGC in adherence assessment may be limited by the subjective account of actual inhaler use. Future clinical pathways may be enhanced further with the application of “e-inhaler” technology in selected “high risk” patients following CGC review and this area requires also detailed prospective study^30^. The use of FeNO in the assessment and management of asthma is gaining prominence within primary care and while the CGC enables the operator intelligently to interpret FeNO readings during a consultation both diagnostically and to aid therapy de-escalation, this was not evaluated in this preliminary analysis^31^. The two cohorts evaluated here represent a group in a PCN with a high deprivation index and in addition satisfied the priority of Health Innovation Manchester STARRS-GM project meeting high-risk criteria for adverse asthma outcomes or suitability for therapy de-escalation as opposed to an unselected asthma population. Nevertheless, it is clear this targeted approach is feasible and the scale of changes suggest beneficial outcomes can be envisaged and a roll out to an additional seven PCN’s is currently underway. As this is a preliminary cross-sectional analysis, we describe the management changes but not the clinical consequences of implementing the changes recommended resulting from the CGC review and a further longitudinal evaluation is planned aiming to measure the impact of this pathway in terms of reduction in SABA use, healthcare utilisation and hospitalisation due to asthma including outcomes in the cohort where de-escalation of therapy occurred.

The CGC was used here in a remote fashion by trained respiratory nurses based in primary care, but future service evaluations will involve use by practice nurses. Such an evaluation will also incorporate and define the training needs of practice nurses and General Practitioners in order to gain competency in the use of the CGC in such a pathway. Already available is an on-line training portal and a test site for users to enter test patient. We do recognise some users may require mentorship support in the first 1–2 clinics. Detailed longitudinal studies are also required to measure the health economic impact of such technology in primary care asthma management alongside any clinical benefits.

We have demonstrated that an end-to-end digital service solution is possible from the recognition of PCNs for prioritisation based on deprivation and/or poor asthma outcomes through to the identification of priority patient groups for review where there is the most gain. The introduction of clinical decision support software in the form of a computer-guided consultation when conducting asthma reviews within primary care is feasible. Not only this, but its use leads to management change in the majority of patients reviewed and the increased implementation of guideline-level standard of care, which is integral to improving patient outcomes and reducing health inequality.

## Supplementary information

Reporting Summary

## Data Availability

The datasets generated during and analysed during the study are not publicly available due to ethical reasons but are available in an anonymised format from the corresponding author on request.
